# Effects of an EMAC Comprehensive Intervention Programme on Poststroke Depression: A Quasi‐Experimental Study

**DOI:** 10.1155/jonm/2615031

**Published:** 2026-10-23

**Authors:** Yijia Luo, Qiqi Ni, Jiayi Hou, Yuyuan Han, Peimeng Teng, Guijuan He

**Affiliations:** ^1^ Nursing School of Zhejiang Chinese Medical University No. 548 Binwen Road, Binjiang District Hangzhou Zhejiang China; ^2^ Nursing Department Sir Run Run Shaw Hospital Affiliated to Zhejiang University School of Medicine Hangzhou Zhejiang China; ^3^ Nursing Department The First Affiliated Hospital to Zhejiang University School of Medicine Hangzhou Zhejiang China

**Keywords:** cognitive behavioral therapy, depression, mindfulness, psychotherapy, quality of life, stroke

## Abstract

**Aim:**

Evaluate the efficacy and safety of EMAC comprehensive intervention programme for poststroke depression patients.

**Background:**

Poststroke depression has a high incidence and significant adverse impacts. Pharmacological treatments for PSD carry certain risks. However, there is currently a lack of a comprehensive, evidence‐based nonpharmacological intervention programme for PSD in China.

**Methods:**

Between May to October 2024, we recruited patients with first‐onset stroke complicated by poststroke depression through convenience sampling from a tertiary hospital in Zhejiang Province. Participants were allocated to the EMAC intervention group and the conventional care control group according to their order of enrolment. This programme incorporated four key components: structured health education, mindfulness‐based stress reduction training, behavioral activation techniques, and standardised cognitive training. ANCOVA was used to compare the primary outcome between groups while adjusting for baseline scores and repeated‐measures analysis of variance was applied to analyze secondary outcomes across the three assessment points.

**Measures:**

The primary outcome of this study was the change in depressive symptoms measured using the HAMD‐17. Secondary outcomes included the five facet mindfulness questionnaire, behavioral activation for depression scale–short form, Montreal Cognitive Assessment, stroke‐specific quality of life scale, and records of adverse event frequency.

**Results:**

Compared with the control group, participants receiving the EMAC programme had significantly lower adjusted HAMD‐17 scores at T1 (10.632 [95% CI, 10.119–11.146] vs. 12.112 [95% CI, 11.611–12.613], *p* < 0.001) and T2 (9.344 [95% CI, 8.663–10.025] vs. 12.053 [95% CI, 11.388–12.718], *p* < 0.001). Secondary outcomes showed significant improvements over time in the intervention group compared with the control group (*p* < 0.05).

**Conclusions:**

This programme can effectively improve the health outcomes of patients and provides valuable evidence and reference for clinical application and dissemination of the intervention.

**Implications for the Patient Care:**

The EMAC programme provides nurses with a practical, evidence‐based protocol for poststroke depression. It combines education, mindfulness, behavioral activation, and cognitive training and may provide a clear and replicable framework for supporting patients with poststroke depression. The observed improvements in depressive symptoms, cognitive function, and quality of life suggest its potential value in clinical nursing practice. Future studies may explore whether selected components of the programme can be adapted to digital platforms to improve accessibility and implementation efficiency.

**Trial Registration:** Chinese Registry of Clinical Trials: ChiCTR2300070335

**Summary**
◦ What does this paper contribute to the wider global clinical community?
• The EMAC comprehensive intervention programme for PSD effectively improved patients’ depressive symptoms, mindfulness, behavioral activation, cognitive function, and quality of life. It provides a reference and basis for clinical promotion.
• The EMAC programme offers potential value for the care and rehabilitation of patients with poststroke depression and may warrant further evaluation in future studies, including investigation of whether selected components can be adapted for digital delivery.

## 1. Introduction

Stroke is characterized by high incidence, disability, recurrence, and mortality rates [1]. In low‐ and middle‐income countries, the incidence and prevalence of stroke are 83.3% and 76.7% [2]. In 2021, there were approximately 93.8 million individuals living with stroke globally, with 11.9 million new cases, and stroke‐related deaths accounted for 10.7% of total global mortality [2]. In China, stroke remains the leading cause of death and disability [3].

Poststroke depression (PSD) is one of the most common and severe complications following stroke. It is typically manifested as persistent low mood, loss of interest, reduced motivation, cognitive slowing, and sleep disturbance, which may further contribute to cognitive impairment and a range of physical symptoms, and in severe cases, even suicidal behavior [4]. The incidence of PSD within 5 years after stroke is estimated to range from 39% to 52% [5]. The mortality risk of patients with PSD is approximately 1.5 times that of patients without depression [6], and their recovery of motor and cognitive functions is significantly poorer, leading to prolonged rehabilitation periods. The development of PSD is multifactorial, involving biological factors (e.g., brain injury, cognitive impairment, and physical disability), psychological factors (e.g., history of affective disorders, specific personality traits, and coping styles), and social factors (e.g., lack of social support and social isolation) [7–10]. Consequently, compared with patients with stroke alone, those with PSD experience more severe conditions, more complex underlying mechanisms, and greater challenges in nursing intervention and ongoing care management [10].

Although antidepressants are commonly recommended for PSD, their use remains controversial due to concerns about adverse events, including an increased risk of intracranial hemorrhage associated with SSRIs [11–13]. Nonpharmacological interventions (NPIs) are scientifically grounded, safe, and noninvasive approaches that can serve as alternatives or complements to pharmacological treatment [14]. Various NPIs have shown promising benefits for patients with PSD, with advantages including minimal adverse effects, high acceptability, and potential for improving depressive symptoms [15–19]. Among these approaches, psychological, behavioral, and cognitive interventions are particularly relevant to the multidimensional nature of PSD because they may address maladaptive coping, reduced behavioral engagement, negative emotional states, and cognitive difficulties that frequently accompany PSD. For example, complementary and alternative therapy (CAT), psychosocial therapy, and cognitive behavioral therapy (CBT) have shown potential benefits for depressive symptoms in patients with PSD [14, 17]. Collectively, these findings suggest that interventions targeting psychological, behavioral, and cognitive processes may provide an important basis for nursing management of PSD.

PSD is influenced by multiple interacting biological, psychological, cognitive, and social factors, and its clinical manifestations are heterogeneous. Accordingly, interventions targeting a single domain may not adequately address the concurrent emotional, behavioral, cognitive, and disease‐management needs of patients with PSD. Previous studies have explored individual psychological or behavioral approaches, including cognitive behavioral interventions, behavioral activation, and mindfulness‐based interventions, with varying effects on depressive symptoms and related outcomes. However, evidence for comprehensive interventions that integrate complementary psychological, behavioral, cognitive, and educational strategies remains limited and inconsistent.

Several comprehensive interventions have been developed for PSD. For example, Van Dijk et al. [20] integrated emotional management, physical activity, and self‐efficacy enhancement into routine nursing care and reported good acceptability and improvements in depressive symptoms. Similarly, Fan and Zhang [21] implemented a multilevel cognitive‐behavioral intervention and observed reduced depression scores at 1 month follow‐up. In contrast, the Stroke Care Optimal Health Program (SCOHP), a broader psychosocial and behavioral intervention, did not demonstrate significant differences in depressive outcomes compared with usual care at 3‐, 6‐, or 12‐month follow‐ups [22]. These inconsistent findings suggest that the optimal organization and integration of intervention components within routine nursing care remain unclear and that the effectiveness of comprehensive interventions may depend on the selection and integration of intervention components, as well as their alignment with the specific needs of patients with PSD. Therefore, an integrated, theoretically informed and evidence‐based approach that incorporates disease‐related education, mindfulness and emotional regulation, behavioral activation, and cognitive strategies may provide a more coherent and clinically applicable framework for nursing management of the interrelated cognitive, emotional, and behavioral challenges experienced by patients with PSD.

To address these gaps, this study was guided by the knowledge–attitude–practice (KAP) theory and synthesised the best available evidence on psychological, behavioral, cognitive, and educational interventions for PSD. The KAP theory proposes that health‐related knowledge provides an important basis for shaping attitudes, while appropriate attitudes can facilitate the adoption of positive health‐related behaviors. Accordingly, interventions that sequentially address knowledge, attitudes, and practices may help patients develop appropriate disease perceptions and translate these perceptions into active self‐management behaviors. Based on this framework and the available evidence, we developed the EMAC comprehensive intervention programme, comprising education (E), mindfulness‐Based stress reduction (M), behavioral activation (A), and cognitive training (C) [23]. The programme was designed not only to address multiple patient‐level needs but also to provide nurses with a structured and reproducible framework for delivering these components within routine care. In the EMAC programme, health education was first provided to improve patients’ understanding of stroke and PSD and enhance disease‐management knowledge. MBSR was then used to promote emotional awareness and regulation, followed by behavioral activation to encourage active engagement in daily and rehabilitation activities. Cognitive Training was incorporated to support cognitive functioning and self‐management. Through this integrated process from knowledge acquisition to emotional adjustment and behavioral engagement, the EMAC programme aimed to alleviate depressive symptoms and improve quality of life (QoL) in patients with PSD. For nursing management, the programme offers a structured approach to coordinating health education, psychological support, behavioral guidance, and cognitive training within an integrated care process, potentially facilitating consistency and continuity in nursing practice. This study subsequently evaluated the effectiveness and safety of the EMAC programme in patients with PSD.

## 2. This Study

### 2.1. Aim

This study employed a quasi‐experimental research method to verify the effectiveness of the EMAC comprehensive intervention programme that we previously developed.

## 3. Methods

### 3.1. Study Design and Setting

This study was originally designed and prospectively registered as a randomized parallel‐controlled trial. However, before participant recruitment commenced, we determined that individual randomization was not feasible within the inpatient ward setting because patients in the same ward frequently interacted with each other and shared rehabilitation activities. Random allocation would therefore have introduced a substantial risk of intervention contamination and compromised intervention fidelity. For this reason, this study was ultimately classified as a quasi‐experimental study. Participants were allocated sequentially according to their time of enrolment. The study was conducted in a tertiary hospital in Hangzhou, Zhejiang Province, China.

The registered protocol also included a self‐developed knowledge questionnaire among the planned outcome measures. However, this measure was not included in the final analyses because its measurement properties were not sufficiently established.

Ethical approval for this study was obtained from the Ethics Committee of Zhejiang Province Hospital of Traditional Chinese Medicine on February 8, 2023 (2022‐KL‐131‐02). This study was prospectively registered in the Chinese Clinical Trial Registry (ChiCTR2300070335) on 10 April 2023. Following trial registration and ethical approval, the intervention manual, investigator training, expert consultation, and study preparation were completed before participant recruitment commenced in May 2024.

### 3.2. Participants

Participants were patients with a first‐onset stroke complicated by PSD. They were recruited through convenience sampling from a tertiary hospital in Zhejiang Province, China, from May to October 2024. The inclusion criteria were as follows: (1) meeting the diagnostic criteria for PSD recommended in the Chinese Expert Consensus on Clinical Practice of PSD [24], (2) having a first‐onset stroke, (3) being hospitalized patients with stable vital signs, (4) having mild to moderate depression, defined as a 17‐item Hamilton depression rating scale (HAMD‐17) score between 7 and 24, (5) being aged 18 years or older, (6) having no severe cognitive impairment, (7) having no severe aphasia, being able to understand and respond appropriately to questions, and being capable of cooperating with training and assessments, and (8) providing written informed consent voluntarily. The exclusion criteria were as follows: (1) presence of severe comorbidities or major physical diseases involving the heart, liver, or kidneys or serious complications, (2) a history of psychiatric disorders, (3) current use of antidepressant medication, participation in other depression‐related interventions, or previous involvement in similar clinical trials, and (4) incomplete data that could affect the evaluation of safety or efficacy.

### 3.3. Sample Size

The sample size was calculated by the calculation formula $n_{1}=n_{2}={\left(Z_{\alpha }+Z_{\beta }\right)}^{2}\times {2\sigma }^{2}/\delta^{2}$ using a two‐sided alpha risk of 5% with a statistical power of 90%. The HAMD‐17 was used as the primary outcome measure. Based on previous study [25], the HAMD‐17 score decreased by 7.03 points in the control group after intervention and by 12.32 points in the intervention group, so the between‐group difference *δ* = 5.29. Assuming a standard deviation of 3.0 for both groups, *α* = 0.05 (two‐tailed), and *β* = 0.10, we obtained *Z*_0.05_ = 1.96 and *Z*_0.1_ = 1.282 from statistical tables. The minimum required sample size for each group was calculated to be 8 participants. Considering a potential 30% attrition rate, the sample size was increased accordingly, resulting in at least 12 participants per group. However, to obtain a more robust sample than the statistical minimum and to reduce the potential impact of attrition and sampling variability, we planned to recruit 24 participants per group. Accordingly, 48 participants were ultimately enrolled. The first 24 participants were assigned to the intervention group, and the subsequent 24 participants were assigned to the control group. After attrition, 41 participants completed the study and were included in the per‐protocol analysis.

### 3.4. Intervention

The intervention group underwent the EMAC comprehensive intervention programme for PSD in addition to routine stroke nursing care. The intervention personnel consisted of three researchers, all of whom had received standardized training. Prior to this study, based on an evidence‐based approach and the KAP model, our research team integrated the best available evidence on nonpharmacological interventions for PSD to develop a preliminary EMAC intervention framework. The programme aimed to break the vicious cycle of dysfunctional cognition, maladaptive behaviors, and negative emotions from a psychological perspective. The initial draft comprised three core components: health education, mindfulness‐based stress reduction (MBSR), and behavioral activation. We invited 10 experts from the fields of neurology, psychology, rehabilitation medicine, and nursing to evaluate the scientific rigor, feasibility, safety, and cost‐effectiveness of the proposed intervention. Based on their feedback, a cognitive training module was added, and the contents of all modules were refined and integrated. The format of the health education component was further optimized, and detailed intervention procedures were supplemented. The final version of the EMAC comprehensive intervention programme was established through group discussions among the research team members.

The EMAC comprehensive intervention programme for PSD consisted of four components: health education (E), mindfulness‐based stress reduction (M), behavioral activation (A), and cognitive training (C). The intervention lasted for 10 weeks, with sessions conducted once per week. The health education component primarily focused on providing knowledge related to stroke and PSD. The MBSR module was modified according to the clinical characteristics, physical tolerance, and safety considerations of PSD patients, following a gradual and progressive training approach. The behavioral activation component included activity monitoring, identifying life domains and personal values, developing an activity list, selecting and ranking activities, and establishing behavioral contracts and daily schedules, as well as consolidating therapeutic gains and preventing relapse. The cognitive training component covered multiple cognitive domains, including visuospatial and executive functions, naming, memory, attention, language, abstraction, delayed recall, and orientation. In this study, cognitive training was implemented using a commercially available mobile application (Future Light‐Fun Brain Training, Version 4.2.0; Beijing Elephant Intelligence Technology Co., Ltd.). The training activities targeted multiple cognitive domains, including memory, attention, processing speed, logical reasoning, calculation, and visuospatial abilities. Details of the cognitive training activities are presented in Table 1.

**TABLE 1:** The EMAC comprehensive intervention programme for poststroke depression.

Duration of intervention	Objective of the intervention	Intervention modalities	Content of the intervention	Homework
The first week	① Introduction of the intervention protocol ② Assessment of baseline conditions to prepare for the intervention	Lecture	**Explanation** (10 min): distribute the “Guideline Manual for the EMAC Comprehensive Intervention Programme for Poststroke Depression” and provide a detailed explanation of the EMAC modules and the specific implementation process. **Assessment** (30 min): collect patients’ baseline data, including evaluation of depressive symptoms, mindfulness and emotional status, behavioral patterns, cognitive function, and quality of life.	—

The second week	**E**: this module provides an overview of the concept and clinical manifestations of stroke, aiming to enhance patients’ understanding of the disease and its potential consequences. **M**: participants are guided through body‐scan exercises to cultivate mindful awareness of physical sensations and the emotional signals transmitted by the body. **A**: this component encourages participants to review their daily activities across a 24‐h period, helping them to identify patterns of avoidance and promote adaptive engagement in meaningful tasks. **C**: this module focuses on memory training designed to strengthen cognitive functioning and support psychological rehabilitation.	Audio, lecture, and discussion	**Health education** (10 min): introducing the concept of stroke, outlining its definition and the principal clinical symptoms. **Body scan** (15 min): participants are instructed to sit back comfortably in a chair or lie supine on the bed with their eyes closed. Following the facilitator’s verbal guidance, they are encouraged to shift their attention gradually across different parts of the body, cultivating awareness of physical sensations. Upon completion of the exercise, participants are invited to share their experiences and reflections on the practice. **Daily monitoring** (20 min): researchers explained the importance and necessity of completing the daily monitoring form. Participants are instructed on how to record their daily activities and corresponding emotional states. The aim is to help them identify the associations between activity engagement and mood fluctuations. **Memory training** (15 min): participants are instructed to select one of the three games—Bomb Disposal Expert, Memory Collector, or Photographic Memory—and progress through the levels from low to high difficulty.	Body scan × 6,memory training × 6

The third week	**E**: introducing the rapid recognition of stroke symptoms and appropriate emergency responses. **M**: participants are guided through mindful breathing exercises to enhance concentration and present‐moment awareness. **A**: participants are guided to reflect on their self‐worth across different areas of daily life and to identify meaningful activities that can be transformed into achievable actions. **C**: participants engage in attention training exercises to enhance their concentration and cognitive control.	Audio, lecture, and discussion	**Health education** (10 min): This session focuses on how to recognise the warning signs of stroke in oneself or family members and provides guidance on where to seek appropriate medical care in the event of a stroke. **Mindful breathing** (15 min): participants are instructed to sit comfortably in a chair or lie supine on the bed, following the facilitator’s verbal guidance to focus their attention on the rise and fall of the abdomen during breathing. Upon completion of the exercise, participants are invited to share their experiences and reflections on the practice. **Developing a list of life domains, values, and activities** (20 min): participants are guided to reflect on what matters most to them within various life domains, including interpersonal relationships, education or career, leisure and interests, physical and spiritual wellbeing, and daily responsibilities. They are then encouraged to translate these personal values into observable, measurable, and specific activities. **Attention training** (15 min): participants are instructed to select one of the three games—Color Match, Quick Response, or One and Only—and progress through the levels sequentially from low to high difficulty.	Mindful breathing × 6, imagination training × 6

The fourth week	**E**: introducing the diagnostic examinations and treatment options for stroke. **M**: participants are guided through self‐acceptance exercises designed to alleviate self‐critical emotions and foster a compassionate attitude towards themselves. **A**: participants are instructed to select activities and rank them according to difficulty, based on their personal circumstances and functional abilities. **C**: conducting logical reasoning exercises.	Audio, lecture, and discussion	**Health education** (10 min): this session explains the medical examinations that patients will undergo immediately after being admitted to hospital with a suspected stroke, as well as the subsequent treatment approaches provided for confirmed cases. **Self-acceptance** (15 min): participants follow the facilitator’s verbal guidance to identify and acknowledge both their strengths and weaknesses, learning to accept themselves and release the control of negative thoughts. Upon completion, participants are invited to share their reflections on the exercise. **Activity selection and ranking** (20 min): participants are asked to choose 15 activities generated from their personal list of life domains, values, and activities, and rank them from 1 (easiest to complete) to 15 (most difficult) according to the perceived level of difficulty. **Logical reasoning** (15 min): participants are instructed to select one of the three games—Number Maze, Zigzag Sudoku, or Spiral Labyrinth—and progress through the levels sequentially from low to high difficulty.	Self‐acceptance × 6,logical reasoning × 6

The fifth week	**E**: introducing to essential aspects of nursing care for patients with stroke. M: participants are guided through a loving‐kindness meditation to cultivate positive emotions and compassion towards others. A: participants are instructed to develop an “assistance contract”, identifying specific situations in which they may need help and learning to actively seek support from others when necessary. C: conducting imagination training.	Audio, lecture, and discussion	**Health education** (10 min): this part will introduce “what kind of nursing care will be provided if one is diagnosed with stroke?” **Loving-kindness meditation** (15 min): patients follow the guided instructions to mindfully experience the care and support from people around them, express gratitude, and offer blessings in return; after the exercise, they are invited to share their feelings. **Develop assistance contract** (20 min): patients imagine which activities from their activity list require help from others and establish an assistance contract; once completed, they need to inform their helpers how to provide support in daily activities. **Imagination training** (15 min): patients choose one of the three games—Panda Chase, Pixel Puzzle, or Line Battle—and progress through levels from easy to difficult.	Loving‐kindness meditation × 6, imagination training × 6

The sixth week	**E**: introducing the risk factors and preventive measures of stroke. **M**: providing guidance on self‐affirmation practice to help patients overcome self‐doubt. **A**: assisting patients in developing a daily activity plan. **C**: conducting arithmetic training.	Audio, lecture, and discussion	**Health education** (10 min): introducing “what are the risk factors for stroke? what measures can be taken to prevent it?” **Self-affirmation** (15 min): patients are asked to sit or lie comfortably and, following the guided instructions, offer themselves an affirming smile while allowing this positive emotion to flow throughout the body; after the exercise, they are invited to share their experiences. **Daily planning** (20 min): patients review the 15 target activities they have previously identified, select one low‐difficulty activity, and incorporate it into their daily routine. **Arithmetic training** (15 min): patients choose one of the three games—Step by Step, Smart Budgeting, or Accumulation—and progress through the levels from easy to difficult.	Self‐affirmation × 6, arithmetic training × 6, implementation plan

The seventh week	**E**: introducing the concept and symptoms of poststroke depression. **M**: providing guidance on seated meditation practice to help patients become aware of their sensations during stillness. **A**: assisting patients in developing a daily activity plan. **C**: conducting reaction‐time training.	Audio, lecture, and discussion	**Health education (10 min)**: introducing “what is poststroke depression? what are its main symptoms?” **Seated meditation (15 min)**: Patients are asked to sit or lie comfortably and, following the guided instructions, bring awareness to their breathing, bodily sensations, surrounding sounds, and thoughts; after the practice, they are invited to share their experiences. **Daily planning (20 min)**: discuss any difficulties encountered during the previous week’s activity plan and collaboratively develop a new plan for the upcoming week based on the patient’s condition. **Reaction-time training (15 min)**: Patients choose one of the three games—Card Master, One‐Man Defense, or Sharp Vision—and progress through the levels from easy to difficult.	Seated meditation × 6, reaction‐time training × 6, implementation plan

The eighth week	**E**: introducing the rapid identification and treatment of poststroke depression. **M**: providing guidance on mindful eating practice to help patients engage their five senses and appreciate the experience of eating. **A**: assisting patients in developing a daily activity plan. **C**: conducting personalised cognitive enhancement training sessions.	Audio, lecture, and discussion	**Health education (10 min)**: introducing “how can family members quickly identify poststroke depression? what treatment options are available for individuals diagnosed with poststroke depression?” **Mindful eating (15 min)**: patients are guided through a mindful eating exercise, asked to adopt a comfortable position, and encouraged to engage their senses of sight, touch, smell, and taste while experiencing the food; after the exercise, they are invited to share their feelings. **Daily planning (20 min)**: discuss any challenges encountered in implementing last week’s activity plan and, based on the patient’s condition, determine whether to include more demanding activities. **Personalised cognitive training (15 min)**: cognitive enhancement exercises are tailored to strengthen the patient’s weaker cognitive domains identified through assessment.	Mindful eating × 6, cognitive training × 6, implementation plan

The ninth week	**E**: introducing the nursing care for poststroke depression. **M**: providing guidance on mindful communication exercises to assist in reintegration into society. **A**: assisting patients in developing a daily activity plan. **C**: conducting personalised cognitive enhancement training sessions.	Audio, lecture, and discussion	**Health education (10 min)**: introducing “what nursing care will be provided if poststroke depression is diagnosed?” **Mindful communication (15 min)**: patients are guided through mindful communication exercises, integrating mindfulness into their interactions; after the practice, they are invited to share their feedback. **Daily planning (20 min)**: discuss any issues encountered during the previous week’s activity plan, and collaboratively develop a new daily activity plan based on the patient’s current condition. **Personalised cognitive training (15 min)**: cognitive enhancement exercises are tailored to strengthen the patient’s weaker cognitive domains identified through assessment.	Mindful Communication × 6, cognitive training × 6, implementation plan

The tenth week	① Consolidate the knowledge acquired. ② Reassess the patient’s condition.	Lecture	**Consolidation** (10 min): recommend high‐quality learning materials; guide the patient in practicing daily mindfulness; review whether the BA has facilitated the completion of a challenging activity; and instruct the patient to engage in daily cognitive training. **Assessment** (30 min): reassess the patient’s condition after the intervention, summarising the progress and gains.	—

The control group received routine stroke nursing care in accordance with the standard clinical nursing practices of the department throughout the 10‐week study period. Routine care included implementation of medical orders, medication education, daily life assistance, psychological care, and dietary guidance. These services were provided by the professional medical workers in the ward as part of routine clinical practice. Routine nursing care was not supplemented with any additional study‐specific psychological, behavioral, mindfulness, or cognitive training. As routine care was delivered according to usual clinical practice rather than as a study‐specific standardized intervention, its frequency and duration were not prospectively quantified.

Contact and outcome‐assessment procedures were otherwise consistent between the two groups. Participants in both groups underwent outcome assessments at baseline (T0), immediately after the 10‐week intervention period (T1), and at the 3 month follow‐up (T2). The same outcome measures and assessment procedures were used at each time point.

### 3.5. Measures

#### 3.5.1. Personal Information Questionnaire

We designed a questionnaire to collect sociodemographic information, including sex, age, marital status, educational level, type of medical insurance, type of stroke, and disease duration. Detailed items are provided in Appendix 1.

#### 3.5.2. Hamilton Depression Scale (HAMD)

This scale was developed by Hamilton and his colleagues in 1960 and remains one of the most widely used clinician‐rated instruments for assessing depression. In this study, the 17‐item version of the HAMD was adopted, which was recommended as a screening and evaluation tool for PSD [17]. The scale comprises five factors: anxiety/somatization, weight, cognitive impairment, retardation, and sleep disturbance. Nine items are rated on a 5‐point scale (0 = none, 1 = doubtful or mild, 2 = mild, 3 = moderate, and 4 = severe), while eight items are rated on a 3‐point scale (0 = none, 1 = doubtful or mild, and 2 = definite). A total score of < 7 indicates no depression, 7–17 indicates mild depression, 18–24 indicates moderate depression, and > 24 indicates severe depression. Previous studies have reported a Cronbach’s *α* coefficient of 0.829 for the HAMD‐17, indicating acceptable internal consistency [26].

#### 3.5.3. Five Facet Mindfulness Questionnaire (FFMQ)

This questionnaire was originally developed by Baer and his colleagues in 2006 to assess dispositional mindfulness in daily life. We employed its Chinese version (Ch‐FFMQ) in this study. The questionnaire comprises 39 items across five relatively independent facets: observing, describing, acting with awareness, nonjudging, and nonreactivity. Responses are rated on a five‐point Likert scale ranging from 1 (“never or rarely true”) to 5 (“very often or always true”), with higher scores indicating greater levels of mindfulness. Cronbach’s *α* coefficient for the total scale was 0.86, while the subscales demonstrated Cronbach’s *α* coefficients ranging from 0.63 to 0.81 [27].

#### 3.5.4. Behavioral Activation for Depression Scale–Short Form (BADS‐SF)

This scale was developed by Manos et al. as a simplified revision of the original BADS and exhibits improved psychometric properties and aligns more closely with the theoretical model of behavioral activation [28]. In this study, the Chinese version of the BADS‐SF was employed. The scale comprises nine items covering two domains: behavioral activation (BA) and behavioral avoidance. Items are rated on a seven‐point Likert scale ranging from 0 (“not at all”) to 6 (“completely”), with items 6–8 (behavioral avoidance) scored in reverse. Higher total scores indicate greater BA and lower behavioral avoidance. The Cronbach’s *α* coefficient for the total scale was 0.82 [28].

#### 3.5.5. Montreal Cognitive Assessment (MoCA)

The MoCA was originally developed by Nasreddine et al. in 2004 and was employed in this study using the widely applied Beijing version for Mainland China. The scale evaluates multiple cognitive domains, including visuospatial and executive function, naming, attention, memory, language fluency, abstract thinking, delayed recall, and orientation, with a total score of 30. For participants with ≤ 12 years of formal education, one point is added to adjust for educational bias. Higher scores indicate better cognitive function, with a cut‐off score of ≥ 26 considered normal. Previous studies have demonstrated excellent reliability, with test–retest reliability of 0.922, inter‐rater reliability of 0.967, and Cronbach’s *α* of 0.752 for the total scale. All individual items correlated with the total score at > 0.60, except for the naming item (*r* = 0.402), and the scale exhibits good criterion and construct validity [29].

#### 3.5.6. Stroke Scale Quality of Life (SS‐QOL)

The SS‐QOL was developed by Williams et al. in 1999 and offers greater specificity compared with other generic quality‐of‐life instruments. The scale comprises 49 items across 12 domains: energy, language, family roles, mobility, mood, personality, social roles, self‐care, thinking, vision, upper extremity function, and work/productivity. Responses are rated on a five‐point Likert scale ranging from 1 (“*completely agree*”) to 5 (“*completely disagree*”), with higher scores indicating better QoL. Previous studies have reported excellent reliability, with test–retest and inter‐rater kappa coefficients ranging from 0.82 to 1.00 [30] and Cronbach’s *α* for content reliability exceeding 0.76.

#### 3.5.7. Adverse Events

The occurrence of adverse events, including falls, palpitations, and dizziness, was monitored and recorded both during the intervention and immediately after its completion.

### 3.6. Data Collection

Data collectors received standardized training and conducted the assessments using a uniform set of instructions to ensure that participants fully understood each question. Upon enrolment, participants completed a baseline demographic and clinical questionnaire. Outcome measures were collected at three time points: prior to the intervention (T0), at the end of the 10‐week intervention (T1), and at the 3 month follow‐up (T2). Data collection was conducted on‐site, with questionnaires administered and returned in person. Participants completed the questionnaires by themselves, supplemented by interviewer assistance when necessary. Completed questionnaires were immediately checked for completeness, and any missing items were promptly addressed by asking participants to provide the information. Data collectors were not blinded to group allocation because of the open‐label nature of the intervention.

### 3.7. Statistical Analysis

Data analysis was performed using SPSS 26.0. Analyses were conducted using a complete‐case approach, and missing outcome data were not imputed. Two‐tailed tests were employed, with *p* < 0.05 considered statistically significant. The Shapiro–Wilk test was used to assess the normality of continuous variables, with *p* < 0.05 indicating a deviation from normal distribution. Continuous variables conforming to normal distribution were presented as mean ± standard deviation, whereas non‐normally distributed variables were reported as median and interquartile range. Between‐group comparisons of continuous variables were conducted using independent‐samples *t*‐tests or nonparametric rank‐sum tests, as appropriate. Categorical variables were described as frequencies and percentages, with between‐group comparisons performed using the chi‐square test or Fisher’s exact test, as applicable. The primary outcome (HAMD‐17 score) was analyzed using analysis of covariance (ANCOVA), with the post‐intervention HAMD‐17 score as the dependent variable, study group as the fixed factor, and the baseline HAMD‐17 score as the covariate. Adjusted mean differences with 95% confidence intervals were reported. Repeated‐measures analysis of variance was used to compare secondary outcome measures between the two groups at each time point (pre‐intervention, post 10 week intervention, and 3 month follow‐up). When the assumption of sphericity was violated, Greenhouse–Geisser corrections were applied. Sensitivity analysis was conducted for the prespecified primary outcome (HAMD‐17) using multiple imputation with predictive mean matching in R (Version 4.6.1). Fifty imputed datasets were generated, and the same ANCOVA models as those used in the primary complete‐case analysis were fitted to the imputed datasets. The resulting estimates were pooled according to Rubin’s rules.

## 4. Results

### 4.1. Characteristics of the Participants

A total of 60 participants were initially recruited from the Rehabilitation, neurology, and neurosurgery departments of a tertiary hospital in Zhejiang Province. Although patients with subarachnoid haemorrhage (SAH) were eligible for inclusion, no eligible patients with SAH were recruited during the study period. Following screening, 48 patients meeting the eligibility criteria and providing informed consent were enrolled in the study, with 24 allocated to the intervention group and 24 to the control group. During the intervention period, two participants were lost to follow‐up in the intervention group (one due to severe pulmonary infection and one due to early discharge) and two in the control group (both withdrew voluntarily). During the follow‐up period, two participants in the intervention group (both due to changes in contact information) and one in the control group (due to changes in contact information) were lost. Ultimately, 41 patients completed the study, including 20 in the intervention group and 21 in the control group, yielding an overall attrition rate of 14.6% (< 20%) (Figure 1).

**FIGURE 1:**
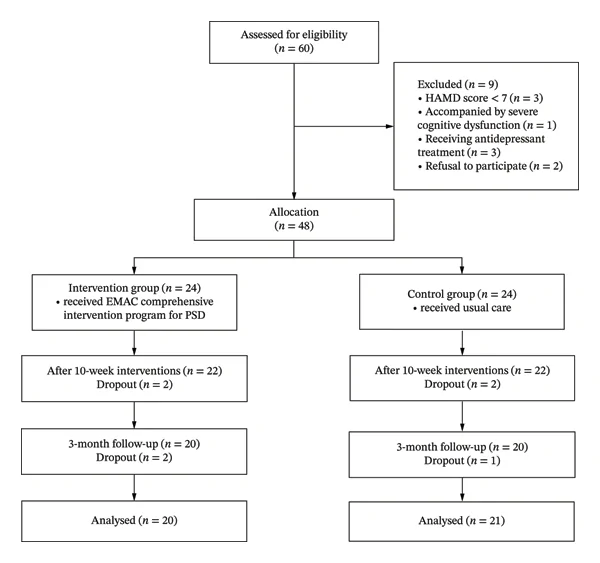
Flowchart of the study.

A total of 48 participants were included in this study, comprising 27 males and 21 females, with a mean age of 68.69 ± 14.17 years. Before the intervention, baseline comparisons were conducted between the two groups. There were no significant differences in baseline characteristics between the groups (*p* > 0.05), indicating good comparability (Table 2).

**TABLE 2:** Comparison of baseline characteristics.

Categorization	Subgroup	Control group (*n* = 24)	Intervention group (*n* = 24)	Statistic	*p*
Gender	Male	12 (50.0)	15 (62.5)	0.762^a^	0.383
	Female	12 (50.0)	9 (37.5)		

Age		71.13 ± 11.338	66.25 ± 16.419	1.209^b^	0.277

Marital status	Single	0 (0)	2 (8.3)	2.623^c^	0.296
	Married	18 (75.0)	19 (79.2)		
	Widowed	6 (25.0)	3 (12.5)		

Educational level	Primary school and below	6 (25.0)	5 (20.8)	−0.950^d^	0.342
	Middle school	8 (33.3)	6 (25.0)		
	High school	7 (29.2)	7 (29.2)		
	College degree or above	3 (12.5)	6 (25.0)		

Medical payment method	Medical insurance	22 (91.7)	23 (95.8)	0.000^e^	1.000
	Self‐funded	2 (8.3)	1 (4.2)		

Type of stroke	Cerebral infarction	22 (91.7)	23 (95.8)	0.000^e^	1.000
	Cerebral hemorrhage	2 (8.3)	1 (4.2)		

Stroke course	4 weeks to 6 months	15 (62.5)	17 (70.8)	−0.606^d^	0.545
	6 months or more	9 (37.5)	7 (29.2)		

HAMD‐17		12.375 ± 3.449	11.417 ± 3.229	0.994	0.326

FFMQ		117.292 ± 10.221	117.458 ± 11.267	−0.054	0.957

BADS‐SF		34.208 ± 3.765	34.958 ± 5.433	−0.556	0.581

MoCA		24.625 ± 1.929	25.000 ± 1.978	−0.665	0.509

SS‐QOL		144.167 ± 17.549	146.083 ± 14.949	−0.407	0.686

^a^Pearson’s chi‐square test.

^b^Independent‐samples *t*‐test.

^c^Fisher’s exact test.

^d^Rank‐sum test.

^e^Continuity‐corrected chi‐square test.

### 4.2. Effect of EMAC Comprehensive Intervention Programme on Patients’ HAMD‐17 Scores

ANCOVA assumptions were met for both time points: the homogeneity of regression slopes was confirmed (T1: *p* = 0.745; T2: *p* = 0.754), residuals were approximately normally distributed, and variances were homogeneous. Compared with the control group, the intervention group had a statistically significant improvement in HAMD‐17 score from T0 to the endpoints at T1 and T2 (T1: 10.400 ± 2.854, *p* < 0.05; T2: 9.150 ± 2.870, *p* < 0.05) (Table 3).

**TABLE 3:** Results of each indicator.

Outcome indicators	T0	T1	T2	P01	P02	P12
HAMD‐17						
Intervention group	12.000 ± 3.146	10.400 ± 2.854	9.150 ± 2.870	< 0.001	< 0.001	< 0.001
Control group	12.524 ± 3.326	12.333 ± 3.168	12.238 ± 2.682	0.471	0.454	0.762
*p* value	0.608	0.047	0.001			
FFMQ						
Intervention group	117.750 ± 11.387	142.950 ± 15.639	143.700 ± 16.040	< 0.001	< 0.001	0.233
Control group	117.048 ± 10.865	117.381 ± 10.632	117.905 ± 10.862	0.85	0.638	0.391
*p* value	0.841	< 0.001	< 0.001			
BADS‐SF						
Intervention group	34.900 ± 5.088	37.300 ± 5.141	38.800 ± 4.491	< 0.001	< 0.001	< 0.001
Control group	34.000 ± 3.962	34.429 ± 3.641	36.610 ± 4.516	0.296	0.332	0.783
*p* value	0.53	0.045	0.002			
MoCA						
Intervention group	25.250 ± 1.888	25.700 ± 1.922	25.800 ± 1.881	0.001	0.001	0.611
Control group	24.619 ± 1.687	24.333 ± 1.560	24.429 ± 1.938	0.03	0.232	0.619
*p* value	0.266	0.017	0.027			
SS‐QOL						
Intervention group	148.100 ± 15.454	175.900 ± 20.604	182.100 ± 17.345	< 0.001	< 0.001	< 0.001
Control group	141.619 ± 16.503	149.524 ± 16.232	161.095 ± 15.678	< 0.001	< 0.001	< 0.001

*Note:* P01 denotes the *p* value for the comparison between baseline and the 10 week post‐intervention assessment; P02 denotes the comparison between baseline and the 3 month follow‐up; and P12 denotes the comparison between the 10 week post‐intervention and the 3 month follow‐up assessments.

According to the ANCOVA findings, the group variable significantly affected the HAMD‐17 scores when adjustment was made for pre‐test state HAMD‐17 scores. In addition, pre‐test state HAMD‐17 scores affected post‐test results. At both T1 and T2, the intervention group showed significantly lower estimated mean scores than the control group (T1: 10.632 [95% CI, 10.119–11.146] vs. 12.112 [95% CI, 11.611–12.613], *p* < 0.001; T2: 9.344 [95% CI, 8.663–10.025] vs. 12.053 [95% CI, 11.388–12.718], *p* < 0.001). The results of the ANCOVA analysis are presented in Tables 4 and 5.

**TABLE 4:** Estimated marginal means (SE) and 95% CIs for HAMD‐17 outcomes across groups and time points.

Outcome	Time point	Intervention group		Control group	
		Estimated EMM (SE)	Estimated difference (95% CI)	Estimated EMM (SE)	Estimated difference (95% CI)
HAMD‐17	T1	10.632 (0.254)	[10.119, 11.146]	12.112 (0.247)	[11.611, 12.613]
	T2	9.344 (0.336)	[8.663, 10.025]	12.053 (0.328)	[11.388, 12.718]

**TABLE 5:** Summary of ANCOVA results for HAMD‐17.

Outcome	Time point		Sum of squares	df	Mean square	*F*	*p*	Partial eta square	Adjusted between group differences	95% CI
HAMD‐17	T1								1.480	[0.762, 2.198]
		Group	22.279	1	22.279	17.394	< 0.001	0.314		
		pre‐test state scores	306.795	1	306.795	239.526	< 0.001	0.863		
		Error	48.672	38	1.281					
	T2								2.709	[1.756, 3.662]
		Group	74.650	1	74.650	33.095	< 0.001	0.466		
		pre‐test state scores	214.647	1	214.647	95.162	< 0.001	0.715		
		Error	85.713	38	2.256					

A sensitivity analysis using multiple imputation was conducted to assess the robustness of the findings to missing outcome data. The results were broadly consistent with those of the primary complete‐case analysis. For HAMD‐17, the adjusted between‐group difference was −1.56 (df 37.90; 95% CI −2.29 to −0.84; *p* < 0.001) at 10 weeks and −2.65 (df 34.08; 95% CI −3.66 to −1.65; *p* < 0.001) at 3 month follow‐up. These findings were consistent with the significant between‐group differences observed in the primary ANCOVA analyses.

### 4.3. Effect of EMAC Comprehensive Intervention Programme on Patients’ FFMQ Scores

Following Mauchly’s test, the FFMQ scores failed to satisfy the assumption of sphericity (*W* = 0.587, *p* < 0.05); consequently, the Greenhouse–Geisser correction was applied. A significant interaction effect was identified between intervention type and assessment time points (*F* = 90.882, *p* < 0.05). The results showed that, after 10 weeks of intervention and at the 3‐month follow‐up, the intervention group’s FFMQ scores were significantly higher than those of the control group (*p* < 0.05). Within the intervention group, the FFMQ scores at 10 weeks post‐intervention and at 3 month follow‐up were both significantly higher than the baseline (*p* < 0.05), but the difference between these two post‐intervention time points was not significant (*p* > 0.05). For the control group, no significant differences in FFMQ scores were observed across baseline, post‐intervention (10 weeks), and follow‐up (3 months) (*p* > 0.05).

### 4.4. Effect of EMAC Comprehensive Intervention Programme on Patients’ BADS‐SF Scores

Following Mauchly’s test, the BADS‐SF scores did not satisfy the assumption of sphericity (*W* = 0.783, *p* < 0.05), thus the Greenhouse–Geisser correction was applied. A significant interaction was found between the intervention type and measurement time points (*F* = 14.867, *p* < 0.05). The results showed that after 10 weeks of intervention and at the 3 month follow‐up, the intervention group’s BADS‐SF scores were significantly higher than those of the control group (*p* < 0.05). Within the intervention group, BADS‐SF scores at both 10 weeks and 3 months post‐intervention were significantly higher than baseline (*p* < 0.05), and the difference between these two post‐intervention time points was also significant (*p* < 0.05). In contrast, no significant differences were observed in the control group’s BADS‐SF scores across all three time points—baseline, 10 weeks post‐intervention, and 3 months follow‐up (*p* > 0.05).

### 4.5. Effect of EMAC Comprehensive Intervention Programme on Patients’ MoCA Scores

Following Mauchly’s test, the MoCA scores did not meet the assumption of sphericity (*W* = 0.831, *p* < 0.05); therefore, the Greenhouse–Geisser correction was utilised. A significant interaction between intervention type and time points was observed (*F* = 6.906, *p* < 0.05). The results demonstrated that after 10 weeks of intervention and at the 3 month follow‐up, MoCA scores in the intervention group were significantly higher than those in the control group (*p* < 0.05). Within the intervention group, both the 10 week and 3 month follow‐up scores were significantly higher than baseline (*p* < 0.05), whereas the difference between these two post‐intervention time points was not significant (*p* > 0.05). Within‐group analysis indicated that MoCA scores in the control group were lower at the 10‐week assessment compared with baseline (*p* < 0.05), however, no significant differences were found when comparing baseline and 3 month follow‐up scores or 10 week and 3 month follow‐up scores (*p* > 0.05).

### 4.6. Effect of EMAC Comprehensive Intervention Programme on Patients’ SS‐QOL Scores

The Mauchly’s test revealed that SS‐QOL scores met the assumption of sphericity (*W* = 0.970, *p* > 0.05). A significant interaction effect was found between the intervention type and measurement time points (*F* = 40.691, *p* < 0.05). Following the 10‐week intervention and at the 3‐month follow‐up, the intervention group’s SS‐QOL scores were significantly higher than those of the control group (*p* < 0.05). Within the intervention group, SS‐QOL scores at both 10 weeks post‐intervention and 3 months follow‐up were significantly higher than baseline (*p* < 0.05), and the difference between these two post‐intervention time points was significant (*p* < 0.05). In the control group, SS‐QOL scores at both 10 weeks post‐intervention and 3 months follow‐up were significantly higher than baseline (*p* < 0.05), with a significant difference also observed between the 10 week and 3 month time points (*p* < 0.05).

### 4.7. Adverse Events

Throughout the intervention period, patients’ vital signs remained stable, and no adverse events such as falls, palpitations, or dizziness were observed.

The summary table of the results is presented in Table 3 and the results of the repeated‐measures ANOVA analysis for the secondary outcome indicators are presented in Table 6.

**TABLE 6:** The results of the repeated‐measures ANOVA analysis for the secondary outcome indicators.

Outcome indicators		df	*F*	*p*	Partial eta square	Diff. (95% CI)
FFMQ	Group effect	1	21.048	< 0.001	0.351	[9.704, 25.007]
	Time effect	1.175	99.922	< 0.001	0.719	T1 to T2 [−15.901, −9.633] T1 to T3 [−16.643, −10.164] T2 to T3 [−1.719, 0.445]
	Group × time interaction	1.175	90.882	< 0.001	0.700	

BADS‐SF	Group effect	1	4.213	0.047	0.098	[0.039, 5.326]
	Time effect	1.567	25.942	< 0.001	0.399	T1 to T2 [−2.139, −0.690] T1 to T3 [−3.167, −1.257] T2 to T3 [−1.413, −0.182]
	Group × time interaction	1.567	14.867	< 0.001	0.276	

MoCA	Group effect	1	4.142	0.049	0.096	[0.007, 2.239]
	Time effect	1.661	1.232	0.293	0.031	T1 to T2 [−0.310, 0.146] T1 to T3 [−0.461, 0.101] T2 to T3 [−0.438, 0.243]
	Group × time interaction	1.661	6.906	0.03	0.150	

SS‐QOL	Group effect	1	12.122	0.001	0.237	[7.524, 28.384]
	Time effect	2	284.904	< 0.001	0.880	T1 to T2 [−20.671, −15.034] T1 to T3 [−29.812, −23.664] T2 to T3 [−11.540, −6.232]
	Group × time interaction	2	40.691	< 0.001	0.511	

## 5. Discussion

The study results suggest that the EMAC comprehensive intervention programme for PSD may improve patients’ depressive symptoms and significantly benefits cognitive function and QoL. Additionally, it may increase patients’ mindfulness and stimulates behavioral activation. The findings suggest that this intervention may yield favorable health outcomes for patients with PSD. However, because MBSR, BA, cognitive training, and health education were delivered as an integrated EMAC programme, the independent contribution of any individual component to these observed improvements cannot be determined in the present study.

### 5.1. The Impact on Patients’ Depressive Symptoms

Participants in the intervention group demonstrated a more pronounced reduction in depressive symptoms relative to the control group. The EMAC comprehensive intervention programme, which included health education, mindfulness‐based stress reduction (MBSR), behavior activation (BA), and app‐based cognitive training, may have facilitated a more holistic understanding of their illness, potentially helping patients address misconceptions about the disease, and cultivate a positive and optimistic outlook on life. By integrating these components into a coordinated nursing intervention, the programme may have enabled nurses to address patients’ psychological, behavioral, cognitive, and educational needs in a more systematic manner. Through the integrated approach, patients may have been encouraged to adopt health‐promoting behaviors and enhance their sense of self‐worth through active engagement. Improvements in cognitive function and behavioral activation observed in this study may also have contributed to the reduction in depressive symptoms [31–35].

### 5.2. The Impact on Patients’ Mindfulness

The study found that participation in the EMAC programme was associated with an upward trend in mindfulness scores among PSD patients, which may partly reflect the integrated mindfulness‐related components of the EMAC intervention, including “body scanning,” “mindful breathing,” “mindful eating,” and “sitting meditation.” These practices may have encouraged patients to focus their attention on present‐moment experiences and may have supported cognitive and emotional regulation. For example, body scanning directs attention to bodily sensations, which may promote present‐moment awareness and emotional regulation [36]. Previous research has suggested that sitting meditation may thicken the cerebral cortex regions responsible for emotion, limiting areas that generate sadness [37]. These results align with another study findings [38]. The intervention group’s FFMQ scores at 3‐month follow‐up were higher than those at 10 weeks post‐intervention and remained elevated, though the difference was not statistically significant (*p* > 0.05). This suggests that patients may have maintained a high level of mindfulness 3 months after intervention, but the lack of professional guidance in mindfulness training post‐intervention may have limited further improvement. This finding highlights the potential importance of continuity of care and structured follow‐up support for maintaining and reinforcing mindfulness‐related skills after the completion of an intensive intervention.

### 5.3. The Impact on Patients’ Behavioral Activation

The results of this study indicate that the EMAC programme was associated with greater behavioral activation among PSD patients. The programme encouraged patients to record daily activities, monitor their content, enjoyment, and importance, identify activity preferences, and engage in discussions to transform valuable life aspects into concrete, manageable activities. Patients are guided to break down chosen activities into manageable steps and develop gradual activity plans to increase confidence in execution. They are also prompted to anticipate potential problems and seek help proactively to overcome obstacles and achieve behavioral activation goals. These behavioral activation strategies may have helped patients increase engagement in meaningful activities and develop greater confidence in managing daily activities. Previous research has suggested that BA may influence neural processes involved in cognitive and emotional regulation, as changes in activation in frontal regions have been observed following BA intervention [39]. These findings provide a possible theoretical explanation for the observed improvement in behavioral activation. Similarly, another study found that BA effectively improves depressive symptoms, consistent with the results of this study [40].

### 5.4. The Impact on Patients’ Cognitive Function

The findings indicate that the EMAC programme was associated with higher post‐intervention cognitive function scores in PSD patients. This improvement may be partly related to cognitive and attentional processes potentially supported by the integrated mindfulness and cognitive‐training components of the EMAC programme. Studies have shown that long‐term practitioners of mindfulness meditation exhibit significantly higher blood flow in the prefrontal cortex, parietal cortex, thalamus, putamen, caudate nucleus, and midbrain compared to nonmeditators [41]. Another research on meditation training reported findings consistent with the potential cognitive benefits of meditation [42]. Additionally, cognitive training in this study may have contributed to cognitive engagement and the maintenance of memory and executive abilities. Previous imaging studies have reported structural changes in brain regions involved in cognitive processing following cognitive training, including the precentral gyrus, prefrontal cortex, and hippocampus, with the prefrontal cortex being closely related to executive functions [43, 44]. These findings may provide a possible theoretical explanation for the observed improvement in cognitive function. A study on cognitive training in PSD patients supports these findings [45]. Notably, while MoCA scores remained elevated at the 3‐month follow‐up, the further increase lacked statistical significance (*p* > 0.05). This may partly reflect the absence of professional guidance and supervision during home‐based cognitive training after the intervention, which may have affected adherence and limited further improvement. In addition, although the control group showed a significant decrease in MoCA scores at 10 weeks, this finding should be interpreted cautiously. The observed change may reflect measurement variability or bias related to the nonrandomized design. Further studies with blinded assessments and larger samples are needed to confirm these findings.

### 5.5. The Impact on Patients’ Quality of Life

This study’s results also suggest that the EMAC programme is associated with higher reported QoL among PSD patients. The core of the MBSR implemented focuses on present‐moment awareness, which may have helped patients develop a more accepting and balanced perspective toward their illness and manage negative emotions, potentially contributing to improved QoL, consistent with previous findings [46]. Additionally, studies by Yokoyama, Xie and others have suggested that BA may reduce functional connectivity between certain subnetworks of the default mode network and the dorsal anterior cingulate cortex, a connectivity negatively correlated with QoL [47, 48]. These findings provide a possible theoretical basis for the contribution of behavioral activation to the QoL improvement observed in our study. The research also combined cognitive training and health education, which may have supported cognitive functioning, treatment engagement, and adaptation to daily life, potentially contributing to improved QoL. Li provided caregiver health education alongside routine care for PSD patients [49], and Li applied modified guided imagery and personalized cognitive training, with results similar to those of this study [50].

From a psychological perspective, PSD often results from poststroke physical dysfunction, combined with a lack of disease‐related knowledge and low engagement in rehabilitation, which can contribute to diminished daily functioning and the emergence of depressive symptoms. Driven by negative emotions, patients tend to withdraw from social activities, resulting in social isolation that can exacerbate depression and creates a vicious cycle involving dysregulated cognition, maladaptive behaviors, and negative emotions. The EMAC comprehensive intervention programme, structured around the KAP model, is designed to help interrupt this cycle among cognition, behavior, and emotion, while showing a positive association with attenuated cognitive impairments. The integrated EMAC programme was associated with reduced depressive symptoms, higher mindfulness levels, improved cognitive function, and greater behavioral activation, all of which may be relevant to patients’ perceived QoL. The programme integrates best existing evidence, expert panel recommendations, and PSD patients’ preferences, ensuring the intervention is scientific, feasible, and appropriate. It offers rich, diverse content delivered through a dedicated PSD EMAC comprehensive intervention manual distributed to each patient, facilitating learning and self‐recording. The intervention employs engaging and autonomous formats including audio and app‐based components which may enhance patient engagement and support the continuation of intervention‐related activities beyond direct nurse–patient contact. These features may improve the feasibility of integrating EMAC into routine nursing and rehabilitation practice and provide a practical basis for its wider clinical implementation.

### 5.6. Limitations and Strengths of the Work, and Recommendations for Further Research

This programme has been constructed by integrating the existing best evidence, absorbing the suggestions of the expert group, considering the needs of PSD patients, and demonstrating the scientificity, feasibility, and suitability of the plan. It has also been verified through quasi‐experimental research methods to ensure the effectiveness of the plan, which is conducive to its further promotion and application.

However, this study still has some limitations. First, the use of convenience sampling at a single tertiary hospital and a nonrandomized controlled design may limit the generalizability of the findings and introduce selection bias and confounding, thereby weakening causal inference. In addition, outcome assessors were not blinded to group allocation, and intervention fidelity was not prospectively assessed, which may have introduced detection bias and made the actual intervention dose difficult to determine. Second, the intervention and control groups differed in contact and attention time, and the frequency and duration of routine nursing care were not systematically quantified. In addition, intervention fidelity was not systematically assessed. Session attendance, completion of weekly home‐based activities, and adherence to the cognitive‐training application were not systematically recorded. Therefore, the actual intervention dose received by individual participants could not be determined, and nonspecific attention effects and differences in usual‐care exposure cannot be completely excluded. These factors should be considered when interpreting the observed between‐group differences. Future randomized studies with attention‐matched control conditions are warranted. Third, no baseline survey was conducted to systematically assess existing PSD management and the needs and experiences of patients and caregivers, and stratified analyses by baseline depressive severity were not performed. Therefore, whether the intervention effects vary according to baseline depressive severity remains unclear. In addition, follow‐up was limited to 3 months, restricting assessment of longer‐term effects. Future multicentre studies with larger and more representative samples and longer follow‐up are warranted. Finally, although HAMD‐17 was designated as the primary outcome, multiple secondary outcomes were assessed, and the possibility of type I error due to multiple comparisons cannot be completely excluded. Moreover, because EMAC was evaluated as an integrated intervention package, the independent contribution of individual components could not be determined. The component‐specific explanations presented in the Discussion should therefore be regarded as possible interpretations rather than evidence of individual component effects. Future studies using appropriate factorial or dismantling designs could further examine the independent and combined contributions of the intervention components. It is recommended that future research could explore whether digital tools, such as mobile applications, could be used to support the implementation of the EMAC programme in clinical settings and whether such adaptations are feasible and effective for patients with PSD.

## 6. Conclusions

This study indicates that the EMAC comprehensive intervention programme for PSD based on the KAP model may improve patients’ depressive symptoms, mindfulness, behavioral activation, cognitive function, and QoL. However, these findings should be interpreted with caution because of the nonrandomised design and the imbalance in intervention‐related contact time between the two groups, which may have introduced bias and limited causal inference. Nevertheless, the findings provide preliminary evidence to inform future research and clinical implementation of comprehensive interventions for PSD. Further randomised controlled trials with comparable contact time between groups are warranted to confirm the effectiveness of the programme.

## Funding

This work was supported by the Health Commission of Zhejiang Province (grant number 2025ZL030).

## Disclosure

Future Light‐Fun Brain Training (Version 4.2.0; Beijing Elephant Intelligence Technology Co., Ltd.) is a commercially available application that can be freely accessed by the public. The research team had no financial, employment, consultancy, or other commercial relationship with the developer. The developer had no involvement in the study design, participant recruitment, intervention implementation, data collection, data analysis, interpretation of the results, or manuscript preparation.

## Conflicts of Interest

The authors declare no conflicts of interest.

## Supporting Information

Additional supporting information can be found online in the Supporting Information section.

## Supporting information


**Supporting Information** Appendix 1 presents the personal information questionnaire used to collect participants’ sociodemographic and clinical characteristics.

## Data Availability

The datasets used and analyzed during the current study are available from the corresponding author on reasonable request.
