# Supplementary material for: Effects of an EMAC Comprehensive Intervention Programme on Poststroke Depression: A Quasi‐Experimental Study
Source: J Nurs Manag. 2026 Oct 23;2026:2615031. doi: 10.1155/jonm/2615031 (PMC13692702; doi:10.1155/jonm/2615031)
Supplement: Supplementary file 1 [file JONM-2026-2615031-s001.docx]

Appendix 1 Personal information questionnaire

1. Gender: □Male □Female
2. Age: ___________
3. Marital status: □Unmarried □Married □Widowed □Divorced
4. Educational level: □Primary school and below □Middle school □High school □College degree or above
5. Medical payment method: □Medical Insurance □Self-funded
6. Type of stroke: □Cerebral infarction □Cerebral hemorrhage □Subarachnoid hemorrhage
7. Stroke course: □4 weeks to 6 months □6 months or more
